# An easy-to-build, low-budget point-of-care ultrasound simulator: from Linux to a web-based solution

**DOI:** 10.1186/s13089-017-0061-4

**Published:** 2017-02-16

**Authors:** Domagoj Damjanovic, Ulrich Goebel, Benedikt Fischer, Martin Huth, Hartmut Breger, Hartmut Buerkle, Axel Schmutz

**Affiliations:** 1 Department of Anesthesiology and Critical Care, Medical Center-University of Freiburg, Faculty of Medicine, University of Freiburg, Hugstetter Strasse 55, 79106 Freiburg, Germany; 2 Information Technology Section, Department of Anesthesiology and Critical Care, Medical Center-University of Freiburg, Faculty of Medicine, University of Freiburg, 79106 Freiburg, Germany

**Keywords:** Ultrasonography, Point-of-care systems, Simulation training

## Abstract

**Background:**

Hands-on training in point-of-care ultrasound (POC-US) should ideally comprise bedside teaching, as well as simulated clinical scenarios. High-fidelity phantoms and portable ultrasound simulation systems are commercially available, however, at considerable costs. This limits their suitability for medical schools. A Linux-based software for Emergency Department Ultrasound Simulation (edus2TM) was developed by Kulyk and Olszynski in 2011. Its feasibility for POC-US education has been well-documented, and shows good acceptance. An important limitation to an even more widespread use of edus2, however, may be due to the need for a virtual machine for WINDOWS^®^ systems. Our aim was to adapt the original software toward an HTML-based solution, thus making it affordable and applicable in any simulation setting.

**Methods:**

We created an HTML browser-based ultrasound simulation application, which reads the input of different sensors, triggering an ultrasound video to be displayed on a respective device. RFID tags, NFC tags, and QR Codes™ have been integrated into training phantoms or were attached to standardized patients. The RFID antenna was hidden in a mock ultrasound probe. The application is independent from the respective device.

**Results:**

Our application was used successfully with different trigger/scanner combinations and mounted readily into simulated training scenarios. The application runs independently from operating systems or electronic devices.

**Conclusion:**

This low-cost, browser-based ultrasound simulator is easy-to-build, very adaptive, and independent from operating systems. It has the potential to facilitate POC-US training throughout the world, especially in resource-limited areas.

**Electronic supplementary material:**

The online version of this article (doi:10.1186/s13089-017-0061-4) contains supplementary material, which is available to authorized users.

## Background

Point-of-care ultrasound (POC-US) has been shown to improve the diagnostic accuracy of the initial clinical assessment of critically ill patients [1]. The use of focused sonography may identify patients with an acute life-threatening condition missed at primary assessment [2]. In the expanding field of POC-US and its wide, but highly user-dependent applicability in emergency situations, adequate training is essential. Hence, training comprises bedside teaching, as well as simulated clinical scenarios, such as advanced life support or trauma resuscitation.

High-fidelity phantoms, as well as portable ultrasound simulation systems are commercially available [3]. However, their application implicates considerable costs, which limits their suitability for resource-limited settings like developing countries as well as for medical schools. University of Freiburg Medical School educates more than 300 third- and fourth-year medical students each year, going through curricular trainings in emergency medicine, as well as anesthesia. To achieve a reasonable student-ultrasound phantom ratio, this would afford the purchase or upgrade of at least three more high-fidelity phantoms only for the curricular training itself. Furthermore, to offer modern concepts of blended learning would require making even more simulators available to students.

In 2011, Kulyk and Olszynski introduced the portable emergency department ultrasound simulator (edus2TM), an easy-to-build, low-cost bedside ultrasound simulator, that allows for integration into high-fidelity simulation scenarios [4]. The machine consists of a laptop computer and a Radio-Frequency Identification (RFID) antenna, hidden in a mock ultrasound probe. RFID tags (cards, points, stickers) attached to phantoms, or standardized patients may then trigger a video sequence, displayed on the laptop and showing the particular ultrasound findings. Thus, pathological POC-US findings can be simulated easily with any phantom or patient. As a result, not only image interpretation on a screen, but also to some extent image acquisition can be trained within real-time settings: the probe has to be held in the right place to retrieve the information (landmarking). The corresponding video files are stored on the laptop computer itself. Unfortunately, the software is LINUX based; this affords a virtual machine to allow its use with WINDOWS^®^. With these restrictions, specific hard- and software has to be used. The concept was published open source by the authors, including technical instructions and a set of sample videos to start with [5]. Another, more up-to-date technology was created by Ostergren. The “Awesome Ultrasound Simulator” is an iOS mobile application, where two smartphones are combined, with one of them displaying the videos, the other serves as a remote control for choosing them [6]. This solution is a freely accessible resource as well, and has successfully been used in the Trauma Resuscitation Using in situ Simulation Team Training (TRUST) study by Petrosoniak and coworkers [7]. It is, however, only available for Apple™ devices, and dependent on a second remote operator to trigger the videos. Following the abovementioned authors’ intention, our aim was to adapt the original edus2TM software toward an HTML-based solution, independent of operating systems or devices, thus making it affordable and applicable in any simulation setting.

## Methods

**Fig. 1:**
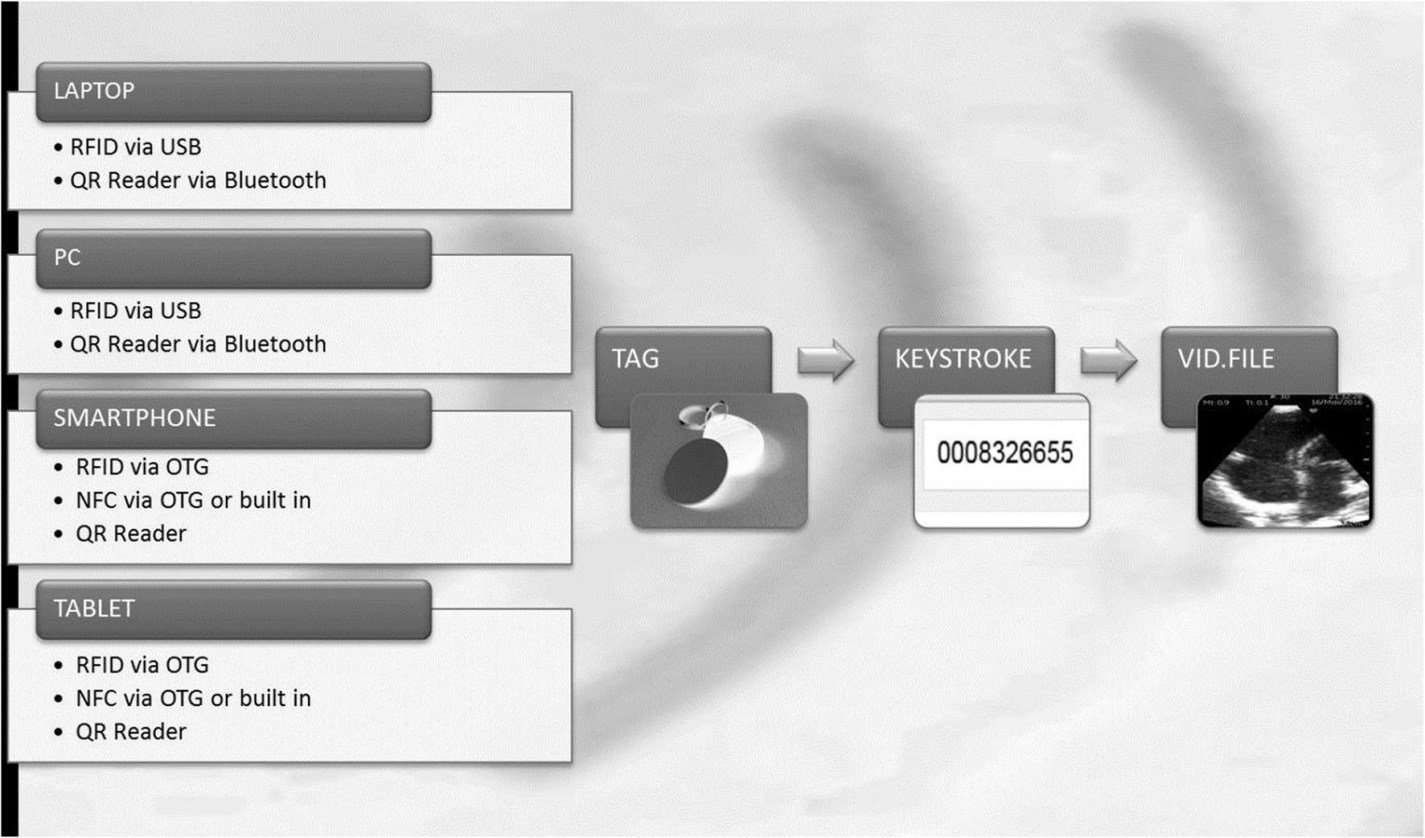
Different devices such as laptop computers, desktop computers/PCs, smartphones as well as tablets can be used to display video files, that is, ultrasound loops, respectively. These are triggered by tags, the content of which is read as a keystroke. *RFID* radio-frequency identification, *USB* universal serial bus, *QR* quick response, *OTG* on the go, *NFC* near-field communication. VID. FILE video of ultrasound loop stored as a separate file

**Fig. 2:**
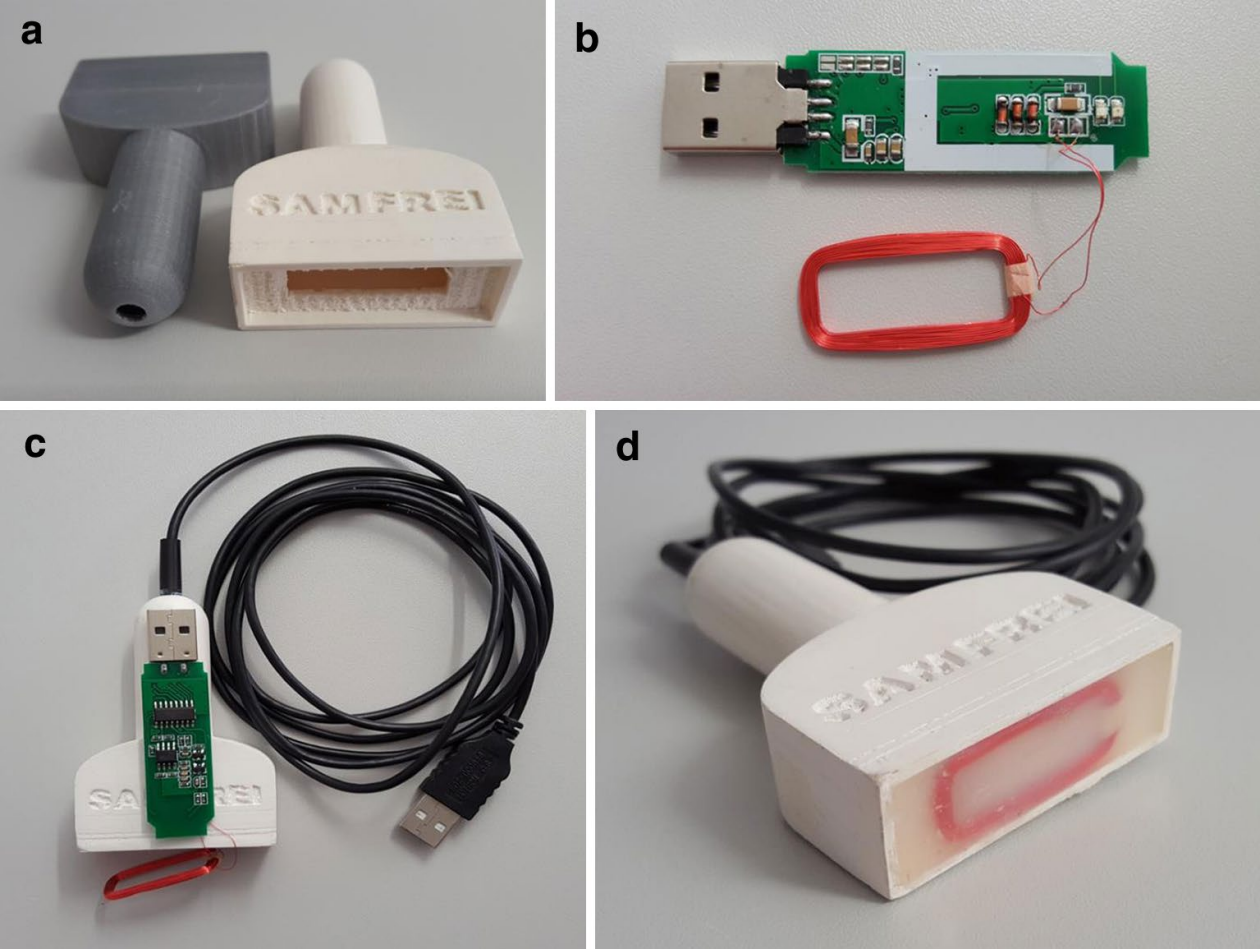
Three-dimensional printed mock ultrasound probe with RFID antenna. **a** Raw material as printed. **b** The partly disassembled RFID antenna, which originally has USB-stick format. **c** How the antenna is built into the housing. **d** The complete probe with USB wire

**Fig. 3:**
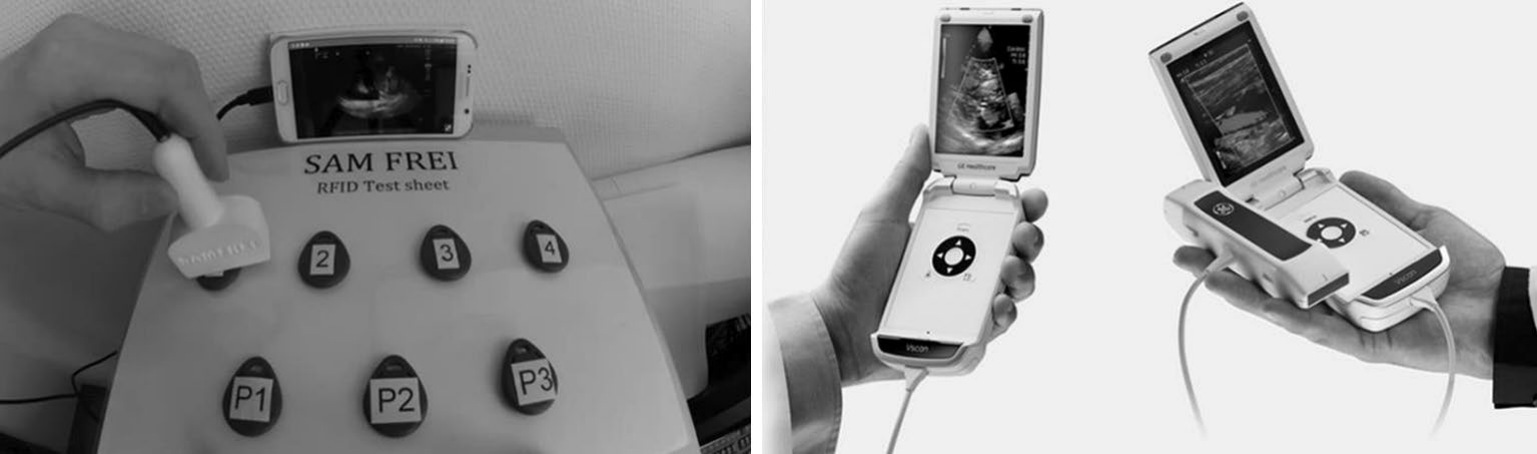
*Left panel* shows the simulator using a mobile phone for the display of a specific ultrasound loop video, *Right panel* shows a commercially available handheld point-of-care ultrasound device (VSCAN DUAL, courtesy General Electric). Please note the three different profile switches, tag P1, P2, and P3 which allow for different findings to be displayed with the same tag *1*, *2*, *3*, or *4*, respectively

Anonymized point-of-care ultrasound video clips from patients, showing individual and unequivocal pathologies have been recorded and stored in a database. Together with our departments’ IT section, we aimed to create a browser-based application written in Hypertext Markup Language (HTML5). This in turn reads the input from a sensor as a keystroke to start displaying ultrasound video clips. Sensors should be RFID antennas, Near-Field Communication (NFC), or Quick Response Codes™ (QR Codes™). The input of the sensor initiates the particular pre-recorded ultrasound video clip on the respective device (e.g., Laptop, Tablet, Smartphone) with any operating system (Mac OS, Android, Windows, Raspian, SUSE, etc.). Access to the pre-recorded videos should be possible wherever they are stored (computer main store, hard drive disk, database server, cloud, etc.). RFID tags were hidden under the artificial skin of a megacode mannequin (ALS Simulator, Laerdal™) or attached to a patient and then masked, as shown in Additional file Digital Content (Additional file 2: SDC video 2). Multiple scans are possible by placement of several RFID tags either on the mannequin or on the patient. Every set of tags can be linked to different profiles, so that several scenarios may be employed using the same hardware. Figure 1 gives an overview of the whole simulation concept and summarizes the different triggers used. As an example, Fig. 2 shows in detail how the RFID antenna was mounted into a self-designed 3D printed mock ultrasound probe (Fig. 2). Video clips provided in the Additional file Digital Content illustrate the concept using a mobile phone (Additional file 1: SDC video 1) or a tablet (Additional file 2: SDC video 2). The different profiles are again chosen by the respective tags, which in SDC video 1 are called P1-P3. Additional file 3: SDC video 3 demonstrates the scanning of an NFC tag directly with a mobile phone, without the need for an additional ultrasound probe. Figure 3 shows a mobile phone which is used as a handheld ultrasound simulator, compared to a commercially available device (General Electric Healthcare, Vscan dual™, courtesy, GE).

An open access version of the simulator application is available at the following URL: https://drive.google.com/file/d/0B7FPzgyHC6cGZHpBa3BFNlpfLVk/view.

You can use the simulator in your own institution and implement own or other pre-recorded ultrasound video clips.

## Results

We successfully created an HTML5-based ultrasound simulator which is independent from the operating system as well as the displaying device, appearing on laptops, personal computer workstations as well as smartphones and tablets with any operating system. It was able to display the corresponding video triggered by RFID tags as well as by Near-Field Communication or QR code detection instantly. The students could replay the ultrasound video clip if necessary. Adding new video clips to the selected file storage worked well, be it in the device itself, a cloud data storage or a video streaming platform.

The total costs for a set of RFID Tags, an RFID antenna, a 3D-printed ultrasound probe, and a USB wire were well below 100 EUR, which is only a minor fraction the cost of commercially available simulated ultrasound systems. Working time for acquisition, adjustment, and review of the video material are not included, however.

## Discussion

The main findings of this study are (a) it is feasible to convert the edus2 software into an HTML browser-based application, (b) this—in combination with a mock US probe—makes ultrasound training and simulation affordable in developing countries, since (c) our software solution may be used on any operating system (Mac OS^®^, Android, WINDOWS^®^, etc.) and on any gadget available (smartphone, tablet, PC, etc.).

Considering the capabilities of our readily available ultrasound simulator at only a fraction of the cost of commercially available solutions, it might be especially relevant for medical education and training in developing countries. These can be remote environments or poor areas in the developing world with a lack of imaging modalities other than ultrasound [8–12]. Of note, acute surges in availability of imaging services can also occur in developed healthcare systems, with emergency ultrasound filling the gap. An impressive illustration of this is the report from the Boston bombings, where POC-US significantly aided in the management of mass casualties [13].

The high adaptability of the system allows for customization according to the requirements of the respective teaching facility. If there is no opportunity to acquire video material by oneself, there are several freely accessible video libraries for this purpose [14].

Due to its low cost and independence of hardware solutions, our simulator allows to be employed for blended learning in our own medical school in a broad manner, which has the potential to spare time and resource. Learners can not only prepare themselves with conventional e-learning material, but also train more aspects of image acquisition, with a unique haptic experience. The latter very much adds to the imaging skills, compared to the sole use of screen-based training choosing videos on the displaying device manually [5]. Given the steep learning curves, quite specific for POC-US, a rapid translation of the clinical skills augmented by such training efforts into clinical routine may be expected [15–17].

When using QR Codes™ as triggers, which can be downloaded and printed out, no additional hardware, i.e., sets of RFID or NFC tags, has to be distributed. Thus, the system can remotely be made available for download. Some minor technical details are subject to our further investigation and development, such as the design of the application surface. Furthermore, strategies for accurate review and quality control of the teaching material have to be implemented. Programming and indexing tags with their corresponding video clips may be time consuming, as it is for any ultrasound video loop editing for educational purposes. As some mobile devices failed to feed battery power to the RFID antennas, the capability of using different triggering solutions provides a useful backup plan. Finally, the sole triggering of a video clip to be displayed does not encompass probe manipulation and image optimization skills as commercially available systems do, which is another field of further technical development for the system. Furthermore, the video output format of ultrasound machines and the anonymizing function varies. Several different file types and codecs may hamper their universal utilization for an ultrasound simulation library. This can easily be overcome using free file converters. Altogether, despite some minor technical issues, we did not want to withhold this important technical advance from publication, since we are convinced, that it will have a considerable impact on worldwide training in POC-US.

## Conclusion

We created a universally available HTML browser-based solution for POC-US simulation at very low cost, which makes ultrasound training and simulation broadly affordable also in developing countries. It was easy to set up and use, running independently from the operating system or displaying device. In medical education, this simulator can be adapted easily to specific demands of the respective setting: undergraduate and postgraduate medical education, simulation, skills training, training of whole POC-US protocols and blended learning. Although the system itself is “low cost,” the technique enables incorporation into high-fidelity simulation, and it enables trainees to learn the indications of ultrasound, learn precise image interpretation, and finally integrate the findings into patient management. We expect our simulator to enhance clinical competence, facilitate health care professionals’ education, and enable research purposes in this important emergency care diagnostic tool.

## Additional files

**Additional file 1.** Mp4, video of cell-phone used as mock handheld ultrasound machine.

**Additional file 2.** Mp4, video of a tablet-pc used as mock handheld ultrasound machine.

**Additional file 3.** Mp4, video with NFC-tags and QR-codes used as ultrasound video triggers.

## Abbreviations

POC-US: point-of-care ultrasound
edus2TM: Emergency Department Ultrasound Simulation
HTML: Hypertext Markup Language
RFID: radio-frequency identification
NFC: near-field communication
QR Codes™: quick response codes™
SDC: supplementary digital content
